# Holocue: A Wearable Holographic Cueing Application for Alleviating Freezing of Gait in Parkinson's Disease

**DOI:** 10.3389/fneur.2021.628388

**Published:** 2022-01-10

**Authors:** Daphne J. Geerse, Bert Coolen, Jacobus J. van Hilten, Melvyn Roerdink

**Affiliations:** ^1^Department of Human Movement Sciences, Faculty of Behavioural and Movement Sciences, Vrije Universiteit Amsterdam, Amsterdam Movement Sciences, Amsterdam, Netherlands; ^2^Department of Neurology, Leiden University Medical Center, Leiden, Netherlands

**Keywords:** freezing of gait, Parkinson's disease, holographic visual cues, unfamiliarity effect, habituation, immediate effect, HoloLens

## Abstract

External visual cueing is a well-known means to target freezing of gait (FOG) in Parkinson's disease patients. Holocue is a wearable visual cueing application that allows the HoloLens 1 mixed-reality headset to present on-demand patient-tailored action-relevant 2D and 3D holographic visual cues in free-living environments. The aim of this study involving 24 Parkinson's disease patients with dopaminergic “ON state” FOG was two-fold. First, to explore unfamiliarity and habituation effects associated with wearing the HoloLens on FOG. Second, to evaluate the potential immediate effect of Holocue on alleviating FOG in the home environment. Three sessions were conducted to examine (1) the effect of wearing the unfamiliar HoloLens on FOG by comparing walking with and without the HoloLens, (2) habituation effects to wearing the HoloLens by comparing FOG while walking with HoloLens over sessions, and (3) the potential immediate effect of Holocue on FOG by comparing walking with HoloLens with and without Holocue. Wearing the HoloLens (without Holocue) did significantly increase the number and duration of FOG episodes, but this unfamiliarity effect disappeared with habituation over sessions. This not only emphasizes the need for sufficient habituation to unfamiliar devices, but also testifies to the need for research designs with appropriate control conditions when examining effects of unfamiliar wearable cueing devices. Holocue had overall no immediate effect on FOG, although objective and subjective benefits were observed for some individuals, most notably those with long and/or many FOG episodes. Our participants raised valuable opportunities to improve Holocue and confirmed our assumptions about current and anticipated future design choices, which supports ongoing Holocue development for and with end users.

## Introduction

Freezing of gait (FOG), an episodic inability to take effective steps (1), is one of the most disabling motor symptoms of Parkinson's disease (PD). Frequently mentioned triggers are turning and initiating walking, while narrow spaces, stress and distraction can also trigger FOG (1). FOG increases the risk of falling, limits mobility, and impacts the quality of life (2–4). It is generally treated with dopaminergic medication or deep-brain stimulation (3, 5). However, there are PD patients where FOG is unresponsive to dopaminergic medication or stimulation (3, 6). FOG can also still be present with seemingly optimal doses (i.e., taking other side effects into account) or may even be induced by medication or stimulation (3, 6). There is thus a need for other FOG remedies to help PD patients with FOG, especially those suffering from FOG in the dopaminergic “ON state.”

External cueing is a well-known means to target FOG. PD patients often use external cues as a compensation strategy to overcome FOG once it has occurred (7). Cues are also used to prevent FOG episodes, mostly using continuous rhythmic auditory, visual, or tactile stimulation for gait synchronization (e.g., rhythmic cues as a pacemaker) (3, 8–10). Although effective (9–11), continuous cueing has adverse side effects, such as developing cue dependency (12, 13), worsening fatigue (8, 14), and demanding more attention (15, 16). These side effects may be reduced with intelligent (8) or assist-as-needed (17) cues, implying cue activation at freeze-prone situations (at the right time) and/or at freeze-prone locations (at the right place).

PD patients who suffer from FOG may benefit more from visual cues than from auditory cues (18), attributable to their stronger action-relevance (19–21), also known as affordances (22). For example, 3D cues like bars or hurdles are action-relevant examples in the sense that they afford stepping over, while 2D cues like stepping stones or stripes taped to the ground primarily afford stepping onto. PD patients seem to have an exaggerated response to such affordances (20, 23) enabling them to bypass defective basal ganglia circuitry (21). While recent studies suggest that action-relevant 3D visual cues may be more effective than 2D visual cues (19, 20, 24), it is accepted that patients' responses to specific types of cues vary strongly (20, 25). This calls for an individual approach with patient-tailored cues to improve their effectiveness for alleviating FOG. A disadvantage of action-relevant visual cueing has long been that they were location-bound. However, recent technological advances have made promising steps toward wearable forms of action-relevant visual cueing, including the laser shoe (26) and headsets with visual displays to augment the real or virtual world with digital visual cues (19, 27, 28).

**Figure 1:**
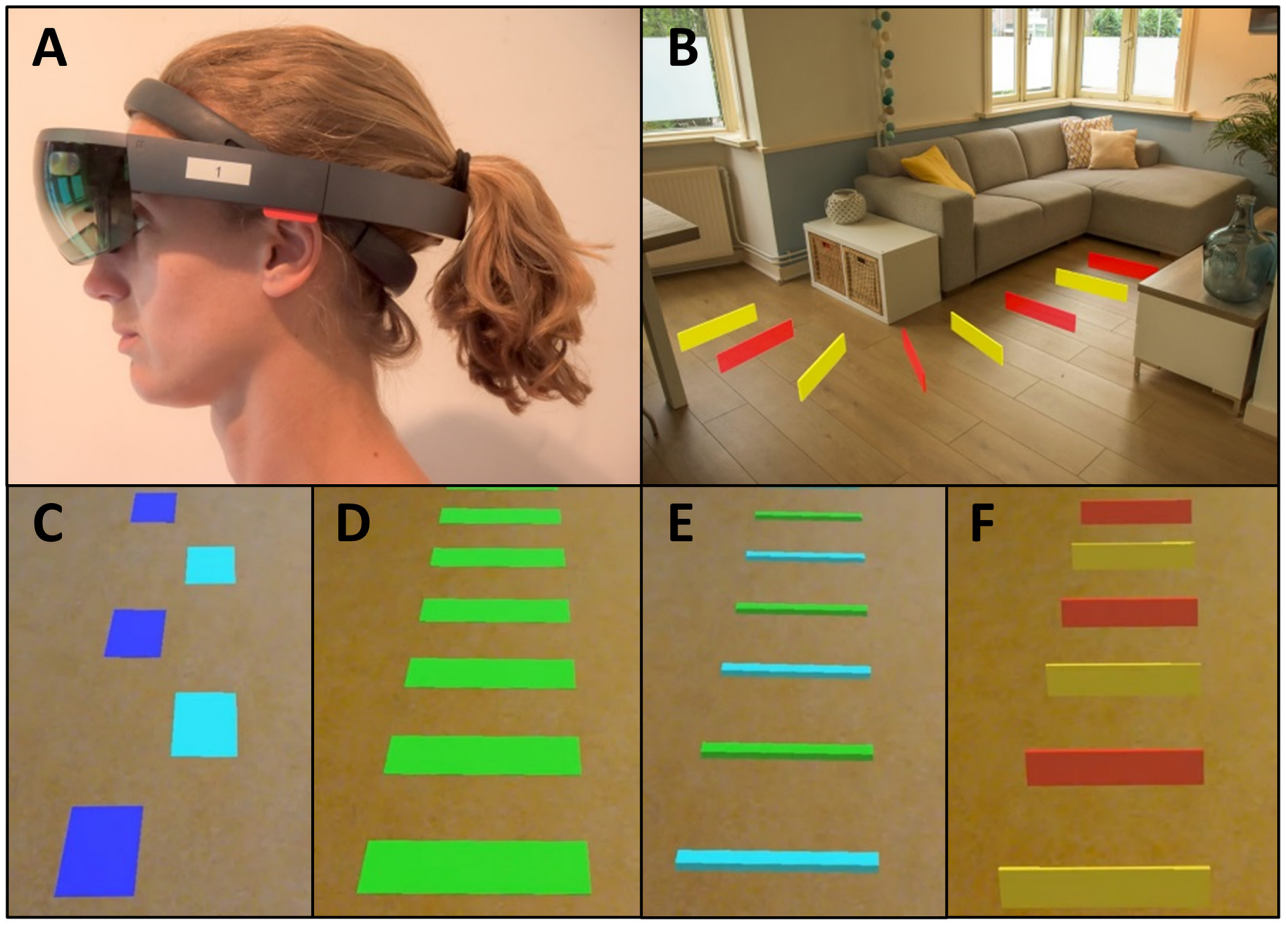
Mixed-reality wearable headset (Microsoft HoloLens 1) **(A)**, with holographic visual cues of Holocue in a home environment **(B)** and a selection of different types of action-relevant holographic visual cues of Holocue in the form of 2D stepping stones **(C)**, 2D zebra stripes **(D)**, 3D beams **(E)**, and 3D hurdles **(F)**.

Inspired by the rapid technological progress for action-relevant visual cueing in free-living environments, we developed a visual cueing application, coined Holocue, for wearable mixed-reality technology (i.e., Microsoft HoloLens 1; Figure 1A). HoloLens is a wearable untethered and non-occluding mixed-reality headset with a holographic display unit through which 2D and 3D holograms can be displayed in the wearer's environment (Figure 1B; see Supplementary Video for an example of 2D holographic visual cues of Holocue in a home environment). HoloLens does not constrain central and peripheral vision of the real world, although the mixed-reality field of view (i.e., the display in which holograms are visible) is much smaller than the field of view of the eyes, and estimated to be about 30° wide and 17.5° high for HoloLens 1. The display resolution is 1,280 × 720 per eye and the pixel density is over 2.5 k light points per radian. HoloLens uses multiple sensors to map its environment and to localize its 3D position in that environment. This location-awareness allows, among other things, for blending holographic content with the wearer's environment (Figure 1B), coined mixed reality.

We use this technology in our Holocue application to position action-relevant 2D and 3D holographic cues onto the walking surface (Figures 1C–F), affording stepping onto and stepping over, respectively. Another advantage of our Holocue application is that cues can be individually tailored: users can pre-select their preferred type of holographic cues (e.g., 2D stepping stones or 3D hurdles; Figures 1C,F) and choose their color, size, and intercue distance. Users can also change the cues when the effect of a particular type of cues is wearing off in order to preserve the potential FOG alleviating effect of Holocue. In the current version of the Holocue application, the wearer can activate the holographic visual cues on-demand through a voice command, for example during a FOG episode to help escape from it or preventatively at or near freeze-prone locations or situations.

The aim of this study is to explore the potential immediate effect of Holocue's on-demand patient-tailored holographic visual cues, as a means to alleviate FOG in free-living environments in PD patients suffering from “ON state” FOG, for whom currently no evidence-based medication or stimulation is available to alleviate FOG (3, 29). Previous studies in controlled lab environments examining the effect of holographic visual cues on FOG showed no demonstrable effect during walking (19, 30) or turning (31), which may be attributable to the overall low occurrence of FOG observed in the lab (30). Another limitation in previous research on wearable cueing devices is the absence of a control condition without the wearable (19, 30, 31), leaving the influence of potential unfamiliarity effects of the wearable (e.g., distraction) on FOG unexplored. Mixed-reality headsets are currently quite heavy (e.g., HoloLens 1 weighs 579 g) and uncomfortable (19) and can therefore divert attention. Distraction in general is known to deteriorate FOG (32–34) and to counteract positive FOG-alleviating effects of visual cues (32). However, when patients get used to the wearable, the potential negative effects of wearing an unfamiliar device on FOG may reduce or disappear. We should thus take (habituation to) wearing an unfamiliar device into account when examining potential FOG-alleviating effects of Holocue. To this end, three sessions were conducted in the current study to examine (1) the effect of wearing an unfamiliar device on FOG by comparing walking with and without wearing HoloLens, (2) habituation effects to wearing an unfamiliar device by comparing FOG while walking with HoloLens over sessions, and (3) the potential immediate effect of Holocue on FOG by comparing walking with HoloLens with and without Holocue. We expect that walking with the unfamiliar HoloLens device increases FOG (unfamiliarity effect), but that this negative effect reduces with habituation over sessions. In addition, we expect overall less FOG with Holocue, with a wide variety in the selected types of cues and their activation, which would validate our design assumptions on the need for patient-tailoring cues and empowerment over their activation. As mixed reality is a rapidly evolving technology, with better headset comfort and field of view coming to the fore, the potential for holographic cueing applications like Holocue for PD patients will grow. Because the development of such applications benefits strongly from user experiences and feedback, the secondary aim of our study is to validate current and anticipated future design choices of Holocue, to examine its acceptability and perceived usability and to discover opportunities for improvement with end-users.

## Methods

### Participants

PD patients suffering from FOG in the dopaminergic “ON state” were recruited from regular clinical care, both inside and outside the Leiden University Medical Center, and via advertisement (e.g., online platforms that facilitate scientific research in PD). In order to be eligible for participation in this study, a participant had to meet all of the following criteria: being 18 years or older, have command of the Dutch language, being diagnosed with PD according to the UK Parkinson's disease Society Brain Bank clinical diagnostic criteria (35), experience FOG in the dopaminergic “ON state” (i.e., experience FOG while the medication is working optimally for other PD-related features). A potential participant who met any of the following criteria was excluded from participation in this study: additional neurological diseases and/or orthopedic problems seriously interfering with gait function, inability to comply with the protocol due to insufficient general fitness or cognitive/communicative inability to understand instructions and participate in the measurement, inability to walk independently. Participant were assessed an hour after medication uptake to ensure they were in the “ON state.” All participants provided written informed consent, and the study was approved by the local Medical Ethical Committee (P18.065).

### Experimental Procedure

**Figure 2:**
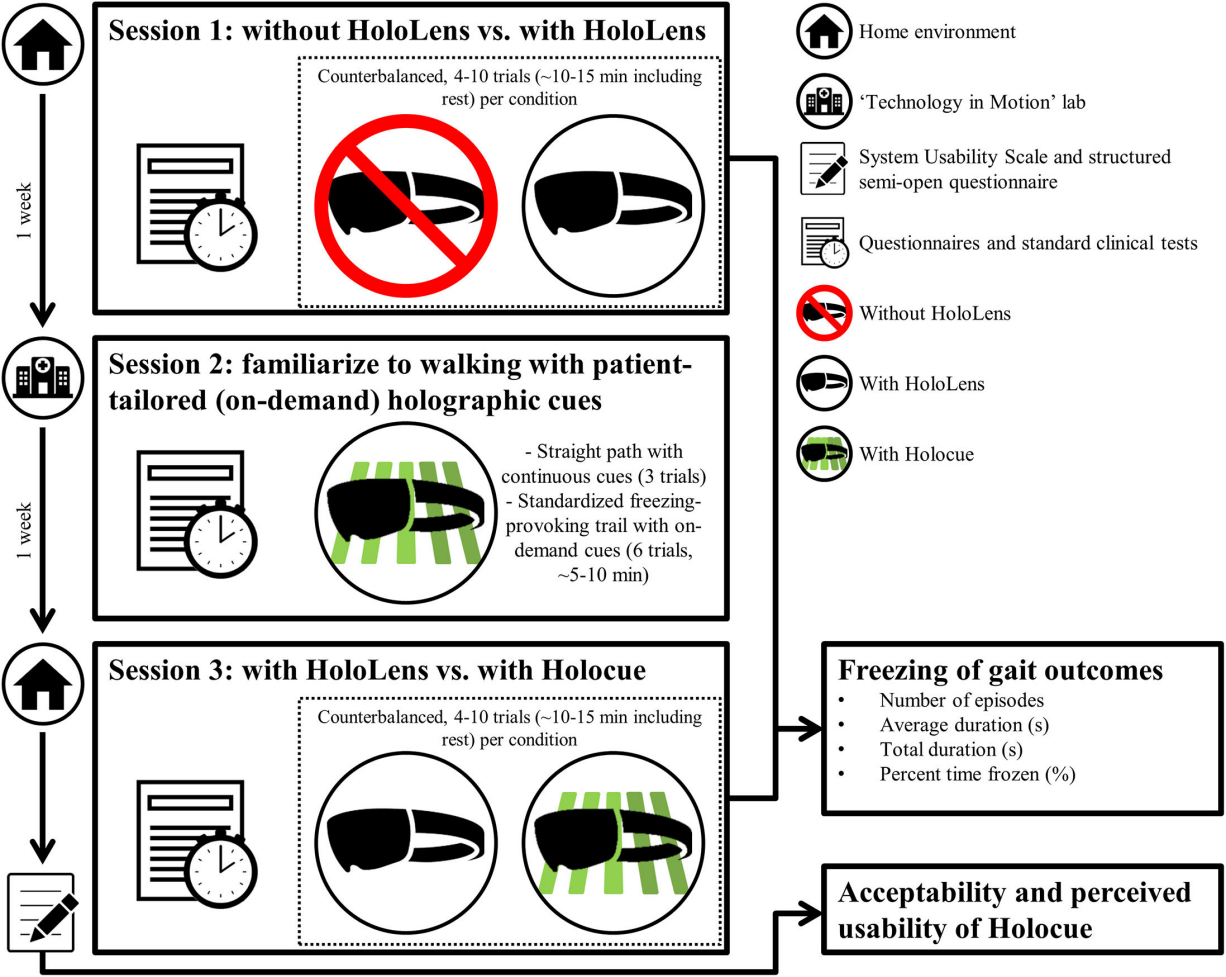
Overview of the sessions (see Supplementary Material 1 for the detailed experimental protocol). Sessions were scheduled one week apart and lasted about 1.5 h.

**Figure 3:**
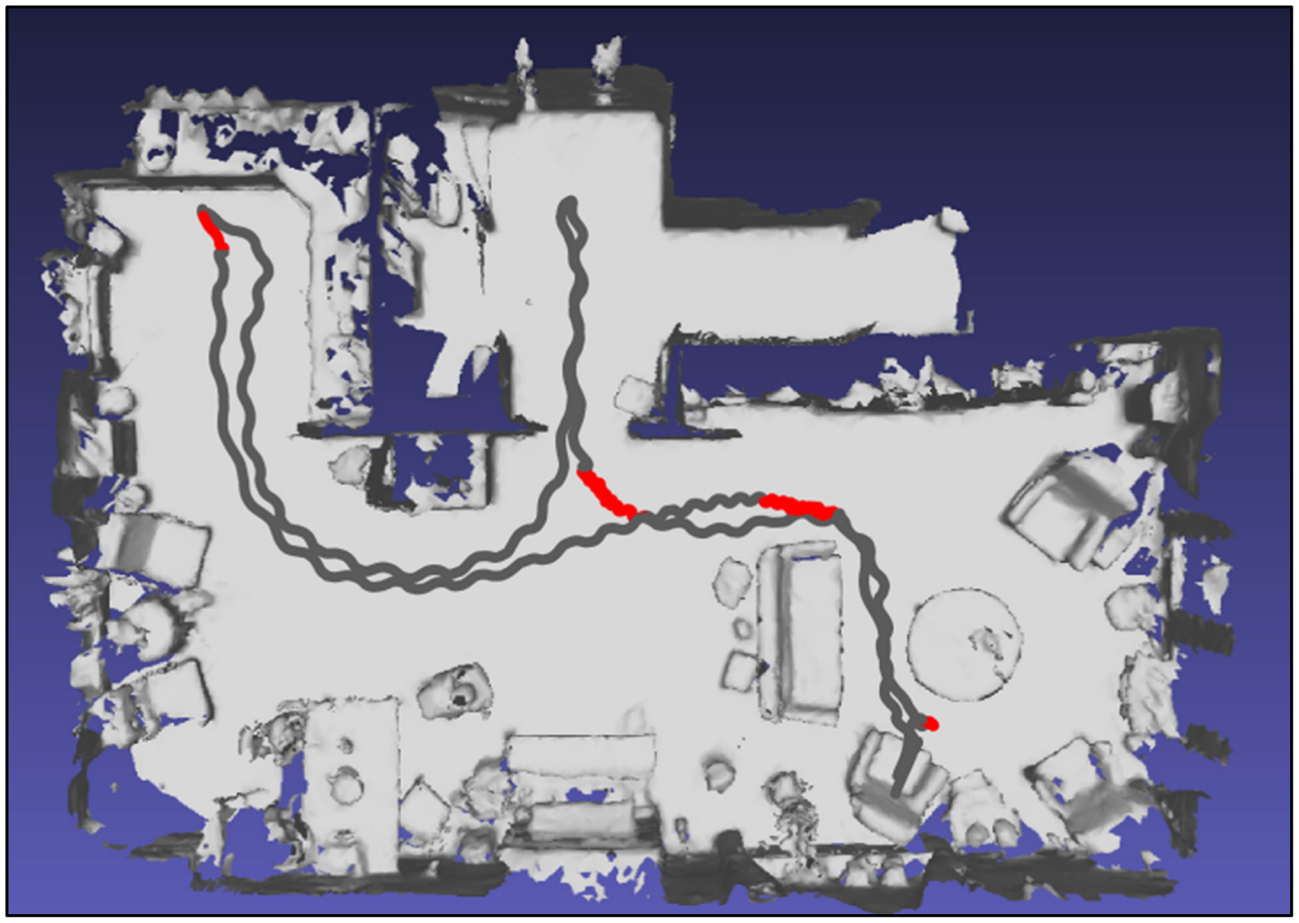
Top view of a participant's home environment as mapped with HoloLens with the route walked based on localization of the HoloLens in the mapped environment (gray trace, representing a single trial with HoloLens without Holocue) onto which the annotated freezing episodes are superimposed with red color.

**Figure 4:**
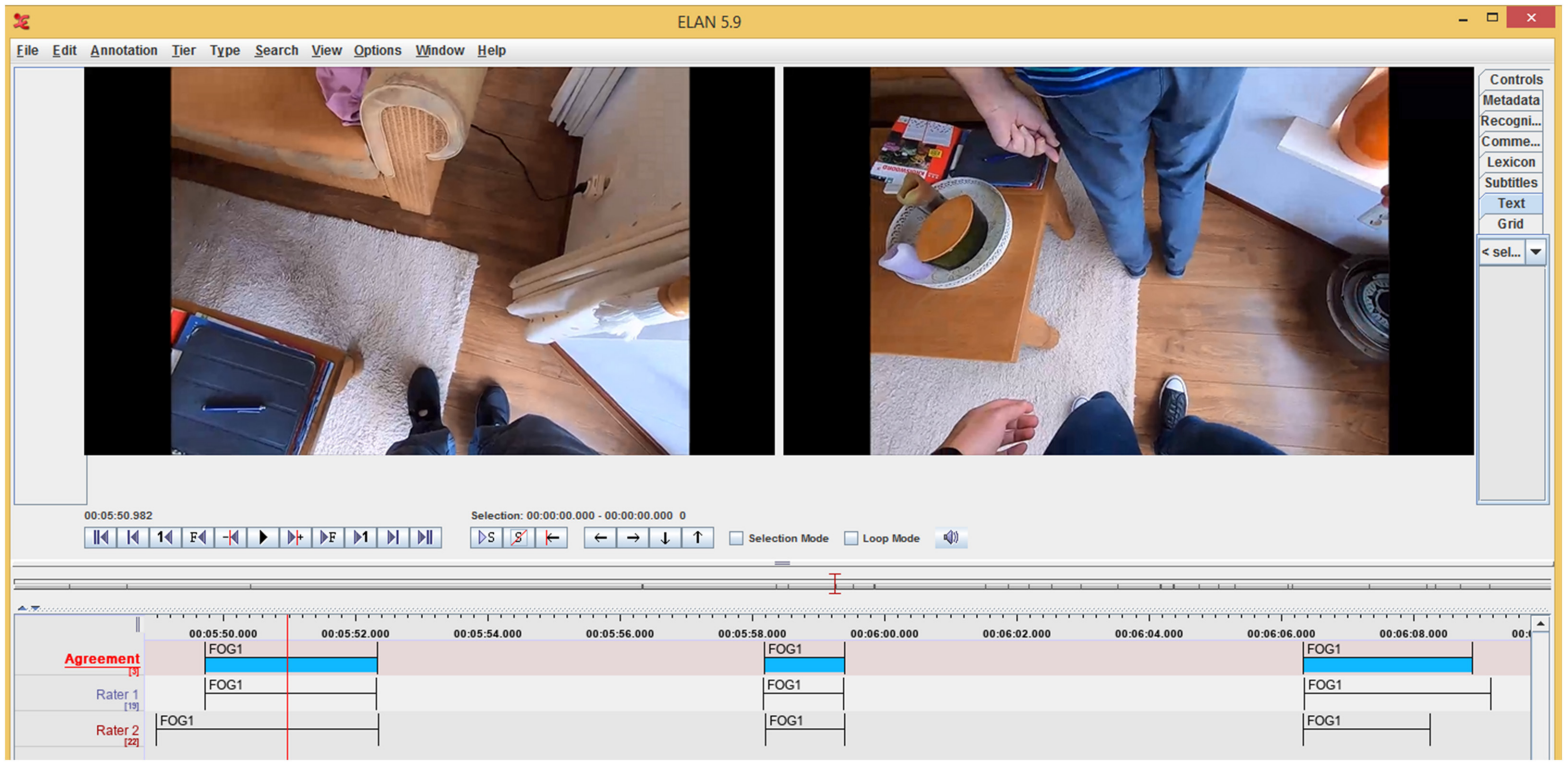
Exemplary annotations of Raters 1 and 2 (bottom two tiers) in ELAN version 5.9 based on videos from GoPro cameras attached to the chest of the participant (left video still) and researcher (right video still). In the top tier, the final FOG annotations (blue patches) are shown after automatically combining annotations of Raters 1 and 2 for time points closer than 200 ms apart the average of the two ratings was taken (cf. the starts and ends of the second FOG annotation in the depicted tiers) or after consensus was reached by discussing time points further than 200 ms apart (cf. time points to the far left and right in the tiers).

This study consisted of three sessions, scheduled 1 week apart at the same time of day. Figure 2 gives and overview of the sessions while the detailed experimental protocol can be found in Supplementary Material 1. Session 1 took place in participants' home environments. To examine the effects of wearing an unfamiliar device on FOG, participants walked a freezing-provoking route (e.g., Figure 3 and Supplementary Video) multiple times with and without wearing the HoloLens (without Holocue) in counterbalanced order. Routes and number of trials per condition differed between participants, but the researcher ensured similar walking routes, number of trials and instructions over the two conditions of a session per participant. Session 2 took place in the “Technology in Motion” lab of the Leiden University Medical Center and was mainly intended to individually tailor the cues of the Holocue application in terms of intercue distance and preferred type of cues (see Figures 1C–F for the types of cues) and familiarize participants to walking with the (on-demand) holographic cues. Session 3 was again a home-based session. During Session 3, participants walked the same route in their house with the same number of trials per condition and with the same instructions as in Session 1. They did so while wearing the HoloLens with and without the Holocue application for patient-tailored (according to Session 2) holographic cues with on-demand cue activation, in counterbalanced order to study habituation effects associated with wearing an unfamiliar device as well as the immediate effect of Holocue on FOG. Walking trials of Sessions 1 and 3 were filmed using two GoPro Hero 7 cameras (30 Hz), one attached to the chest of the participant and one attached to the chest of the researcher, both focusing on the feet of the participant (Figure 4) for offline annotation of FOG episodes. The GoPro videos were synchronized using a synchronization sound generated with MATLAB R2017a.

Questionnaires and standard clinical tests were divided over the three sessions to lower the burden on the participant (Figure 2), and included the following validated assessment instruments: the motor examination of the Movement Disorders Society Unified Parkinson Disease Rating Scale (36) and Hoehn and Yahr stage (37) to assess disease severity, the Montreal Cognitive Assessment (38) to assess cognitive abilities, and the New Freezing of Gait Questionnaire (39) to assess the severity of FOG.

To evaluate acceptability of the Holocue application, a short questionnaire on usability (i.e., System Usability Scale) (40) and a structured semi-open standardized questionnaire were filled out after completion of Session 3 (Figure 2; see Supplementary Material 2 for the full questionnaire), in the absence of the researcher to avoid gratitude bias (41).

### Data Analysis

All videos were annotated for FOG afterwards using ELAN version 5.9 [(42, 43); Figure 4], each by two independent raters. We considered three types of FOG: the trembling type of FOG, the akinetic type of FOG and festination. Independent raters annotated the start and end of FOG episodes, using the definitions of Gilat (44). A total of 1,167 FOG episodes were annotated by Rater 1 (DJG), while Rater 2 (comprising one of three research assistants) annotated 1,188 episodes. The percentage agreement between raters for time points (sampled at 30 Hz) was high (93.9 [93.0–94.5]%). Cohen's kappa, an agreement measure that takes the agreement occurring by chance into account (45, 46), for time points was 0.67 [0.61–0.71], indicating a substantial agreement between raters (47). The discrepancy between the two agreement measures relates to the relatively low prevalence of FOG in the time series, yielding a relatively low positive agreement (59.6 [49.0–68.1]%, due to variation in the exact FOG onset and offset time points) and a very high negative agreement (95.4 [94.1–96.2]%). Overall, between-raters agreement outcomes improved with more FOG. The intraclass correlation coefficient for absolute agreement [ICC_(A, 1)_] (48) for percent time frozen, demonstrating a stronger agreement between raters than total FOG duration in (49), was good to excellent for all sessions and conditions [ICC_(A, 1)_ ≥ 0.878] (50). This indicates that although time points did not exactly match, FOG episodes were overall well-annotated.

After independent annotation of the FOG episodes, the annotations were merged. If start or end did not differ more than 200 ms, which was the case for 380 (40.8%) start time points and 575 (61.8%) end time points, the average was taken of both raters (e.g., start and end of second FOG annotation in the depicted tiers in Figure 4). Otherwise, the raters had to discuss until they reached consensus (e.g., time points to the far left and right in Figure 4). This was also the case when only one of the two raters annotated a FOG episode (about 20% of the episodes annotated, 257 out of 1,167 for Rater 1 and 237 out of 1,188 for Rater 2) or if there was a disagreement about the type of FOG. Hereafter, the number and duration (average duration, total duration and percent time frozen) of FOG episodes were determined. Percent time frozen was defined as the total duration of FOG episodes divided by the total time of the walking trial (i.e., minus sitting) times 100%.

### Statistical Analysis

The number and duration (average duration, total duration and percent time frozen) of FOG were compared with paired-samples *t*-tests between conditions within sessions to examine the effect of wearing an unfamiliar device (Session 1: without HoloLens vs. with HoloLens) and the immediate effect of Holocue on FOG (Session 3: with HoloLens vs. with Holocue) and between sessions to examine habituation effects to the HoloLens (walking with HoloLens in Session 1 vs. Session 3). These comparisons were also evaluated with Bayesian hypothesis testing (51, 52) using JASP (Version 0.9.10) (53). This analysis quantifies how much more likely the data support the alternative hypothesis (FOG differs between conditions or sessions) compared to the null-hypothesis (FOG does not differ between conditions or sessions), reported as the Bayes factor BF_10_ (alternative/null). In line with Jeffreys (51), we regard BF_10_ values between 1 and 3 as anecdotal evidence, values between 3 and 10 as moderate evidence, values above 10 as strong evidence for the alternative hypotheses. The inverse of these values suggests evidence for the null-hypothesis. Independent-samples *t*-tests and one-way ANOVAs were used to examine if the order in which the counter-balanced conditions were administered influenced difference scores between conditions and sessions.

## Results

### Participants

Twenty-four PD patients suffering from FOG in the dopaminergic “ON state” participated in this study. The sample size allowed for fully counterbalancing the order of the conditions (2 × 2) of this within-subject study design. Participants were (mean [range]) 67.0 [55–76] years, of which 15 males and 9 females. The average disease duration was 15.4 [7–31] years and participants had a levodopa equivalent daily dose of 953 [75–2,230] mg (15 participants with deep brain stimulation). Participants had a Movement Disorder Society version of the Unified Parkinson's Disease Rating Scale motor score of 40.0 [15–59], a Hoehn and Yahr stage of 2.2 (see UPDRS motor score for mean [range] example): [2–3], and a Montreal Cognitive Assessment score of 25.6 [12–30]. A total of 1,156 annotated FOG episodes were included in the analyses after consensus between raters, with an average number of 12.0 [1.3–31.5] episodes per condition per participant and an average duration of 4.8 [0.9–21.3] s. These scores are in line with the high scores of 18.8 [11–26] observed for the New Freezing of Gait Questionnaire. Clinical test scores and FOG outcomes per condition and session are presented as Supplementary Data.

### Does the HoloLens Have an Unfamiliarity Effect (Session 1)?

**Figure 5:**
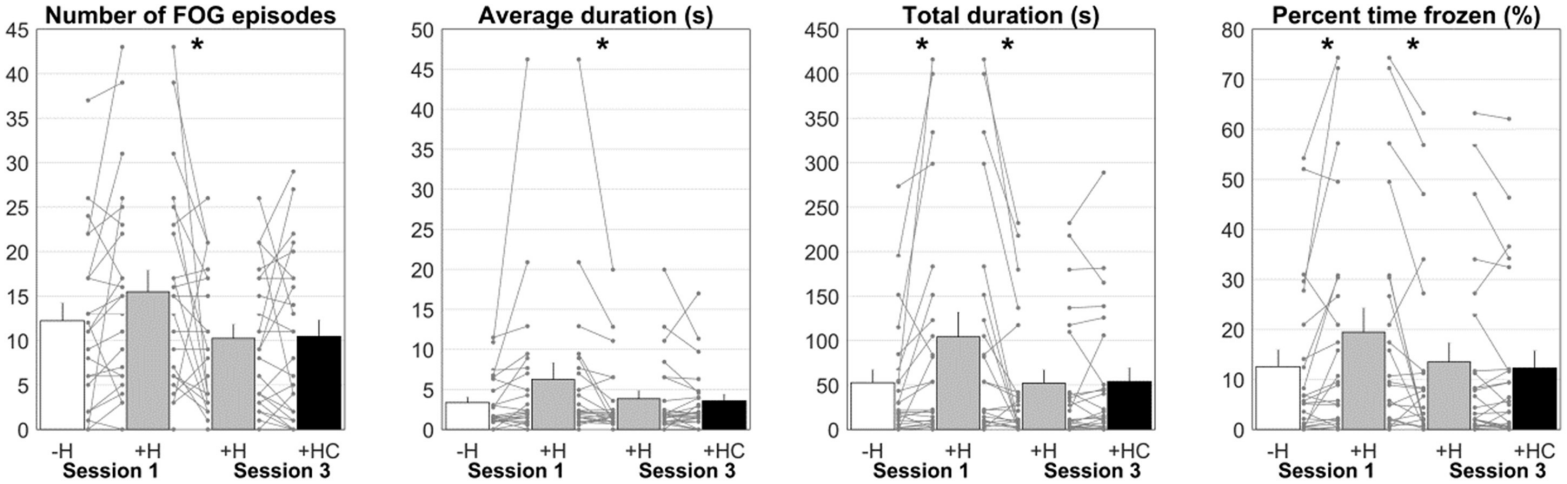
Number of FOG episodes, average duration, total duration, and percent time frozen for Session 1 without HoloLens (–H; white bar) and with HoloLens (+H; gray bar) and Session 3 with HoloLens (+H; gray bar) and with Holocue (+HC; black bar). Asterisks indicate significant comparisons. Each line represents a participant.

One participant was excluded from the analyses of Session 1 (without HoloLens vs. with HoloLens) because walking routes differed markedly between conditions (participant 2). For the remaining 23 participants, total FOG duration was significantly longer [104.2 ± 132.8 s vs. 52.5 ± 70.2 s, *t*_(22)_ = 2.57, *p* = 0.017, BF_10_ = 3.11] and percent time frozen was significantly higher [19.5 ± 23.1% vs. 12.6 ± 16.1%, *t*_(22)_ = 2.66, *p* = 0.014, BF_10_ = 3.62; Figure 5] for walking with than without HoloLens. For the number of FOG episodes [15.5 ± 11.6 vs. 12.2 ± 9.5, *t*_(22)_ = 2.00, *p* = 0.058, BF_10_ = 1.17] and the average FOG duration [6.2 ± 10.0 s vs. 3.3 ± 3.3 s, *t*_(22)_ = 1.79, *p* = 0.087, BF_10_ = 0.86; Figure 5] a tendency toward significance was found, with more and longer FOGs for walking with HoloLens. The order in which the conditions were administered did not influence these results (*p* ≥ 0.265). Wearing the unfamiliar HoloLens device thus increased FOG.

### Does the Unfamiliarity Effect Habituate (Session 1 vs. Session 3)?

Habitation to wearing HoloLens was examined by comparing walking with HoloLens in Session 1 to walking with HoloLens in Session 3. The same participant as in Session 1 was excluded from the analysis. Number of episodes [15.5 ± 11.6 vs. 10.1 ± 7.8, *t*_(22)_= 2.29, *p* = 0.032, BF_10_ = 1.89], average duration [6.2 ± 10.0s vs. 3.6 ± 4.8s, *t*_(22)_= 2.14, *p* = 0.044, BF_10_ = 1.46], total duration [104.2 ± 132.8 s vs. 49.6 ± 72.8 s, *t*_(22)_= 3.33, *p* = 0.003, BF_10_ = 13.62] and percent time frozen [19.5 ± 23.1% vs. 13.1 ± 19.0%, *t*_(22)_= 2.70, *p* = 0.013, BF_10_ = 3.92] all decreased significantly from Session 1 to Session 3 (Figure 5). The order in which the conditions were administered did not influence these results (*p* ≥ 0.402). Habituation to wearing the HoloLens diminished FOG.

### What Is the Immediate Effect of Holocue on FOG (Session 3)?

No systematic differences were found in Session 3 for walking with HoloLens with and without Holocue in terms of the number of FOG episodes (10.3 ± 7.7 vs. 10.5 ± 9.0, *t*_(23)_ = 0.15, *p* = 0.884, BF_10_ = 0.22), average FOG duration [3.8 ± 4.8 s vs. 3.6 ± 4.0 s, *t*_(23)_= 0.52, *p* = 0.608, BF_10_ = 0.24], total FOG duration [52.1 ± 72.3s vs. 53.9 ± 75.1s, *t*_(23)_ = 0.31, *p* = 0.762, BF_10_ = 0.22] and percent time frozen [13.5 ± 18.7% vs. 12.3 ± 17.0%, *t*_(23)_= 1.19, *p* = 0.246, BF_10_ = 0.40; Figure 5]. The order in which the conditions were administered did not influence these results (*p* ≥ 0.155). However, as expected, the settings of the cues (i.e., selected type of cues, distance between cues, percentage activation of cues) differed considerably between participants (Table 1). So did the immediate effect of Holocue on FOG and the subjective reports on its experienced effect, which did not always match the objective results (Table 1, Figure 5). Positive effects of Holocue were mainly seen in participants with long and/or many FOG episodes (i.e., participants 2, 6, 11, 18, and 20; Table 1). The percentage activation of cues was overall high (64.5 ± 21.0%), without an apparent relation to its effect on FOG (Table 1). Whereas systematic immediate effects of Holocue on FOG were absent, the manner in which Holocue was used and its effects were quite variable, with apparent demonstrable objective and subjective benefits for some.

**Table 1:** Individual results of the settings of the cues, the immediate effect of Holocue on FOG, and answers to some questions from the structured semi-open standardized questionnaire from Session 3.

	**Cues**			**Number of episodes**		**Average duration (s)**		**Total duration (s)**		**Percent time frozen (%)**		Structured semi-open standardized questionnaire^*^			
**Participant**	**Type of cues**	**Intercue distance (cm)**	**% Activation**	**With HoloLens**	**With Holocue**	**With HoloLens**	**With Holocue**	**With HoloLens**	**With Holocue**	**With HoloLens**	**With Holocue**	**System Usability Scale**	**Less FOG episodes**	**Shorter FOG episodes**	**Walk better**
1	2D-S	70	64.7	15	14	1.8	2.9	27.3	40.0	7.8	9.4	57.5	5	3	3
2	2D-S	60	15.3	13	11	8.4	4.6	109.5	50.3	22.9	11.6	42.5	4	4	4
3	3D-B	65	75.4	3	5	1.0	1.5	3.1	7.4	0.5	1.2	57.5	3	3	2
4	2D-Z	60	63.1	2	0	2.0	0.0	4.0	0.0	1.9	0.0	65.0	2	2	2
5	2D-S	45	54.4	3	0	0.9	0.0	2.8	0.0	1.1	0.0	57.5	–	–	–
6	2D-S	65	78.4	26	13	1.6	1.8	41.7	22.7	11.2	4.9	50.0	4	4	4
7	3D-B	65	75.1	4	2	0.7	1.4	2.9	2.8	0.8	0.6	42.5	1	2	1
8	3D-H	60	57.2	2	1	1.1	1.0	2.1	1.0	0.7	0.3	57.5	3	3	4
9	2D-S	60	53.8	1	0	0.7	0.0	0.7	0.0	0.2	0.0	37.5	3	3	2
10	2D-S	45	95.1	6	2	1.6	1.5	9.7	3.0	2.0	0.7	42.5	3	2	5
11	2D-S	50	100	9	16	20.0	11.3	179.7	181.4	56.9	46.3	50.0	2	3	4
12	2D-S	55	13.7	11	11	3.6	4.0	39.2	44.0	11.7	12.0	–	1	1	1
13	2D-Z	40	76.0	4	8	2.4	1.9	9.7	15.2	4.4	5.6	2.5	1	1	1
14	2D-S	70	72.5	4	1	1.9	1.7	7.4	1.7	2.0	0.4	35.0	2	2	2
15	2D-S	50	45.9	8	6	1.4	2.1	11.1	12.8	3.2	3.4	57.5	2	2	2
16	2D-Z	50	79.9	11	27	1.9	3.9	21.1	105.8	8.1	11.8	30.0	1	1	1
17	2D-Z	60	59.2	21	29	6.5	4.8	136.6	138.5	27.2	36.6	–	4	4	5
18	2D-S	45	66.5	17	17	12.8	9.7	217.8	164.9	47.0	34.2	47.5	2	2	3
19	2D-Z	55	73.7	0	4	0.0	3.1	0.0	12.4	0.0	3.6	55.0	4	3	4
20	2D-S	60	71.4	21	4	1.5	2.2	32.2	8.8	6.7	1.4	55.0	3	3	2
21	2D-Z	75	51.9	9	21	0.7	1.0	6.4	19.9	2.1	6.4	62.5	1	1	3
22	2D-S	70	80.9	17	22	2.1	2.1	35.7	45.5	8.3	9.4	–	3	3	3
23	2D-S	40	81.8	21	17	11.1	17.0	232.1	288.8	63.2	62.1	35.0	3	3	3
24	2D-S	35	41.1	18	20	6.5	6.3	117.2	125.7	34.0	32.4	50.0	4	4	4

*FOG, freezing of gait; 2D-S, 2D stepping stones; 2D-Z, 2D zebra stripes; 3D-B, 3D beams; 3D-H, 3D hurdles*.

* *1, totally disagree, 5, totally agree*.

*Participants who demonstrated a decrease in number (3 or more episodes) and/or average duration (more than 1 s) of FOG episodes are highlighted in gray*.

### User-Experiences and Recommendations for Future Holocue Development

**Figure 6:**
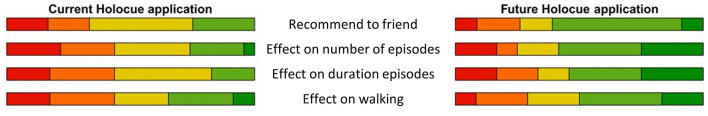
Scores on comparable questions on the structured semi-open standardized questionnaire for the current and future Holocue application (after improvements in headset comfort and field of view). The width of the colored sections is equal to the percentage response from very negative (red) to very positive (dark green). Scores were overall more positive for the future application with anticipated improvements in headset comfort and field of view for holographic content.

As expected considering the small mixed-reality field of view limiting visibility of nearby holographic cues and the bulkiness of the HoloLens 1 headset, all participants scored the usability of the current Holocue application below the SUS-cutoff of 68 (47.1 [2.5–65.0]) (40). Participants were rather positive about a future Holocue application when being pictured improved comfort of the headset and functionality (see Supplementary Material 2 for individual scores on the closed-ended questions), as could also be appreciated from comparing questions that appeared in both current and future Holocue application sections (Figure 6; Supplementary Material 2). Recommendations from participants were to include auditory cues (e.g., metronome or music) and improve the usability of the cues for assisting with turning. In total, 17 out of 20 participants (not all participants filled in all questions) recommended continuing with the development of the Holocue application.

## Discussion

The aim of this study was to explore the potential immediate effect of Holocue on “ON state” FOG in free-living environments in PD patients. We also focused on potential unfamiliarity and habituation effects associated with wearing an unfamiliar device. Although overall less FOG with Holocue was expected, no systematic immediate effect of Holocue on FOG was found in Session 3 with its counterbalanced conditions of walking with and without Holocue. As expected, wearing the unfamiliar HoloLens device significantly increased FOG compared to walking without HoloLens (counterbalanced conditions of Session 1). This unfamiliarity effect on FOG significantly reduced over sessions to the level of walking without HoloLens in Session 1 (Figure 5), indicating that participants habituated to wearing the device. When only two sessions would have been performed, one with HoloLens and one with Holocue, we could have erroneously concluded that Holocue is effective. After all, the total FOG duration and percent time frozen were both significantly lower when walking with Holocue in Session 3 compared to walking with HoloLens in Session 1 (54.0 ± 76.8 s vs. 104.2 ± 132.8 s, *t*_(23)_= 2.87, *p* = 0.009, BF_10_ = 5.39; 12.3 ± 17.4% vs. 19.5 ± 23.1%, *t*_(23)_= 2.78, *p* = 0.011, BF_10_ = 4.54). Including proper control conditions (i.e., without HoloLens in Session 1 and with HoloLens in Session 3) prevented us from arriving at such false-positive conclusions on the immediate effect of Holocue. Likewise, with a hypothetical single-session design contrasting the number and duration of FOG between Holocue and not wearing HoloLens conditions, we could have arrived at the false-negative conclusion that Holocue would have a detrimental effect on FOG. These actual and hypothetical examples thus not only emphasize the need for sufficient habituation to unfamiliar devices, but also testify to the need for research designs with appropriate control conditions when examining the potential effect of unfamiliar wearable cueing devices.

Next to the experimental design with appropriate control conditions, another strong point of this study was that it was performed in participants' homes under realistic settings. A large number of 1,156 FOG episodes were observed, with clear evidence for unfamiliarity and habituation effects. Many studies on cueing and FOG are performed in controlled laboratory environments and it is known that participants exhibit less FOG in these situations (30, 54). This is also what we noticed during Session 2 in the lab. No to very few and short FOG episodes were observed per participant, making it difficult, if not impossible, to determine unfamiliarity effects associated with wearing the HoloLens and potential immediate effects of Holocue. In addition, it is important to test new assistive cueing devices, like Holocue, in realistic free-living environments where they are intended to be used, as the effect in a controlled lab environment may be very different. A final strong point of this study was the timing of measurements. Most patients suffering from FOG exhibit less FOG with medication (5). Therefore, most studies on FOG remedies are performed under unrealistic settings, namely at moments when medication uptake is delayed or in the “OFF state.” Although wearable cueing applications could certainly also work to remedy “OFF state” FOG (26), we focused explicitly on patients suffering from dopaminergic “ON state” FOG after their regular medication uptake or stimulation to ensure being in an “ON state.” This not only ascertained a realistic setting for testing the immediate effect of Holocue, but also would have a bigger impact because there are currently no evidence-based medication or stimulation solutions available to alleviate FOG for patients with “ON state” FOG. All in all, in the current study, both the home environment, the type of participant and the “ON state” timing of measurements led to more FOG episodes as well as realistic environments and situations for testing immediate Holocue effects.

Although Holocue did not systematically reduce FOG on a group level, objective (shaded rows in Table 1) and subjective improvements were observed for individual participants. As expected, the settings of the cues (i.e., selected type of holographic cues, distance between cues, percentage activation of cues) differed considerably between participants (Table 1). Cues were mainly activated continuously or preventatively at or near freeze-prone locations, possibly leading to less FOG episodes as in participants 6 and 20. However, participants with long FOG episodes (i.e., participants 2, 11, and 18; Table 1) also seemed to benefit from Holocue. This may be explained by the rather small field of view of HoloLens 1, which limits visibility of nearby holographic cues. Thereby, cues are not distracting and not action-relevant unless the wearer looks down. Participants with longer FOG episodes are expected to make the effort to look down and interact with the cues during an episode instead of before an episode, reducing the duration, but not the number of episodes. The variation observed in the use of Holocue within and between participants in combination with the resultant mixed effects of Holocue on FOG, warrants future studies on its merit. Such studies could benefit from a longer practice and habituation time for participants to find out what type of holographic cues and way of activation works best for them to alleviate or prevent FOG in order to avoid such explorations when examining its effect.

Because the development of applications such as Holocue benefits strongly from user experiences and feedback, we further aimed to validate our assumptions on design choices and to discover opportunities for improvement with end-users. In the Introduction, various requirements for effective cueing devices were mentioned, among which action relevance. Participants' responses to the questionnaire items indeed showed that the action relevance of visual cues was deemed important, with a slight preference for stepping onto cues (e.g., 2D stepping stones; Figure 1C) over stepping over cues (e.g., 3D hurdles, Figure 1F; see also Supplementary Material 2). This preference also became apparent from the selected types of holographic cues (Table 1). With respect to patient-tailoring the cues, participants responded positively to the options to choose the type of cues, color, size, and distance between cues (Supplementary Material 2). However, some participants did not prefer visual cues and wanted auditory cues, either alone or in combination with visual cues (Supplementary Material 2). Barthel et al. (26) and Bunting-Perry et al. (54) already demonstrated that not all PD patients suffering from “ON state” FOG benefit from visual cueing.

Mentioned internal opportunities for Holocue improvement mainly comprised modality of cues and patient-tailoring cues. In the current version of the Holocue application the choice was limited to four pre-made types of visual cues. In a future Holocue application, users should be able to adjust more settings, including opting for auditory cues, to better tailor the cues to their needs and desires. Furthermore, future studies should test more settings (e.g., walking with both 2D and 3D cues, varying intercue distance) per individual to better understand gait-modifying and FOG-alleviating effects of various cue characteristics and assess the practical feasibility of the Holocue application (e.g., monitor adverse events, such as falls, and explore the possibilities for independent use). Participants saw potential in Holocue, but indicated that mixed-reality headsets need to improve with respect to comfort and mixed-reality field of view. Participants indeed stated that they had trouble with stepping onto or over cues (see Supplementary Material 2), for which unnatural head postures were required to see the nearby holographic cues during the targeted step or crossing maneuver. As such, the small mixed-reality field of view thus limited the action relevance of holographic cues and their immediate effect on FOG, which could also have played a role in previous studies showing null effects with related technology and cueing applications (19, 27, 30, 31). Interestingly, mixed-reality field of view and wearer comfort have allegedly already strongly improved with the latest generation of mixed-reality headsets, such as HoloLens 2 and Magic Leap 1 and 2. These technological advancements offer external opportunities for Holocue improvement, which will likely increase the usability, patient-friendliness, experienced usefulness and potential efficacy of Holocue, especially when combined with the valuable internal opportunities for improvement we discovered with potential end-users.

## Conclusion

Holocue, with its patient-tailored action-relevant holographic cues, had no systematic immediate effect on “ON state” FOG in the home environment in PD patients. Nevertheless, Holocue holds promise for alleviating FOG because of (1) objective and subjective benefits for some participants (i.e., those with long and/or many FOG episodes), (2) current and anticipated future design choices being validated by end-users, and (3) identified internal (i.e., better patient-tailoring, modality of cues) and external (i.e., better mixed-reality headsets to improve comfort and action relevance) opportunities for improvement. Finally, our study testifies to the importance of controlling for unfamiliarity and habituation effects (as these demonstrably affected the number and duration of FOG episodes) in order to prevent drawing false-positive or false-negative conclusions regarding the effect of new wearable cueing technology.

## Data Availability Statement

The original contributions presented in the study are included in the article/Supplementary Material, further inquiries can be directed to the corresponding author/s.

## Ethics Statement

The studies involving human participants were reviewed and approved by Medisch Ethische Toetsingscommissie Leiden-Den Haag-Delft (P18.065). The patients/participants provided their written informed consent to participate in this study. Written informed consent was obtained from the individual(s) for the publication of any potentially identifiable images or data included in this article.

## Author Contributions

DG and MR: conceptualization and writing–original draft preparation. DG, BC, JH, and MR: methodology and writing–review and editing. BC: software. DG: formal analysis, investigation, and visualization. MR: supervision and funding acquisition. All authors have read and agreed to the published version of the manuscript.

## Conflict of Interest

The authors declare that the research was conducted in the absence of any commercial or financial relationships that could be construed as a potential conflict of interest. After this study was accepted for publication, VU signed a collaboration agreement with Strolll.co as part of a joint ongoing EUreka Eurostars project (115506 MAGIC CUE, start date December 1, 2021), including transfer of IP and technology related to holographic cueing from VU to Strolll.co.

## Supplementary Material

Supplementary Video Video of a participant walking with HoloLens with and without the holographic visual cues of Holocue in a home environment. The participant was asked to walk at comfortable walking speed and first walk to the kitchen (where he should move a cup on the counter), then walk around the coffee table and finally walk back to the starting point. As apparent in the video, time to completion and the number of experienced freezing episodes differed markedly between the two conditions. The participant has consented to the publication of the video.

Supplementary Material 1 Experimental protocol in detail.

Supplementary Material 2 The structured semi-open standardized questionnaire and individual scores on the closed-ended questions for the current (PART I) and future (PART II) Holocue application.

Supplementary Data Clinical test scores and number of freezing of gait (FOG) episodes, average FOG duration, total FOG duration and percent time frozen per condition and session.
