# Supplementary material for: Holocue: A Wearable Holographic Cueing Application for Alleviating Freezing of Gait in Parkinson's Disease
Source: Front Neurol. 2022 Jan 10;12:628388. doi: 10.3389/fneur.2021.628388 (PMC8784874; doi:10.3389/fneur.2021.628388)
Supplement: Supplementary Material 1 — Experimental protocol in detail. [file Data_Sheet_1.pdf]

Supplementary Material 1

1 Experimental protocol

This study consisted of three sessions of about 1.5 hours, scheduled one week apart at the same time of day. Sessions 1 and 3 took place in participants' home environments. Session 2 took place in the 'Technology in Motion' lab of the Leiden University Medical Center. Questionnaires and standard clinical tests were divided over the three sessions to lower the burden on the participant, and included the following validated assessment instruments: the motor examination of the Movement Disorders Society Unified Parkinson Disease Rating Scale [1] and Hoehn and Yahr stage [2] to assess disease severity, the Montreal Cognitive Assessment [3] to assess cognitive abilities, and the New Freezing of Gait Questionnaire [4] to assess the severity of FOG.

1.1 Session 1 – home environment

Session 1 aimed to examine potential unfamiliarity effects on FOG associated with wearing the HoloLens (without Holocue functionality). At the beginning of this home-based session, the researcher discussed with the participant when and where the participant experiences FOG in their house using the Characterizing Freezing of Gait Questionnaire [5]. Subsequently, participants walked a route set out by the researcher in their own home and visited locations that were identified by the participant as being freeze prone (e.g., walking through doorways, turning in narrow spaces; [6]). To examine the effects of wearing an unfamiliar device on FOG, participants walked the routes with and without wearing the HoloLens (without Holocue functionality) in counter-balanced order.

The researcher explained the route before the first walking trial and also reminded the participant of the route during the first few trials and in between if necessary, but no practice trials were performed. The participants walked the route 4-10 times per condition, the exact number depending on the length of the route and the physical condition of the participant. This could be combined with motor or cognitive dual tasks to increase the likelihood of FOG [7,8]. The instruction given to the participants was: 'Walk the predetermined route at comfortable walking speed with/without cognitive dual task.' The cognitive dual task was either naming words which started with a specific letter or serial subtraction by seven. Routes and number of trials differed between participants, but for every participant the instructions, the number of trials and the number of trials with a dual task were the same for each of the two counterbalanced conditions, except for participant 2. Walking routes differed markedly between conditions and this participant was therefore excluded from the analyses of Session 1.

Measurements took about 10-15 minutes per condition, including rest. The researcher always walked closely behind the participant for safety reasons. Walking trials were filmed using two GoPro Hero 7 cameras (30 Hz), one attached to the chest of the participant and one attached to the chest of the researcher, both focusing on the feet of the participant (Figure 4) for offline annotation of FOG episodes. The GoPro videos were synchronized using a synchronization sound generated with MatLab R2017a.

1.2 Session 2

Session 2 was a laboratory session, which was mainly intended to individually tailor the cues of the Holocue application in terms of intercue distance and preferred type of cues. The distance between the cues was adjusted to the step length of the participant walking at a self-selected comfortable walking speed without wearing the HoloLens, as measured with the Interactive Walkway [9,10], an instrumented 8-meter walkway for markerless full-body 3D motion registration. Intercue distance was set to the average step length observed over three trials, rounded to the nearest 5 cm with a minimum of 35 cm considering the size of some of the cues (i.e., the zebra stripes, Figure 1D). Participants then selected their preferred type of holographic cues, choosing from 2D stepping stones (Figure 1C), 2D zebra stripes (Figure 1D), 3D beams (Figure 1E) or 3D hurdles (Figure 1F). Participants walked with each type of cues at least once on an 8-meter walkway to determine the preferred type. Using the so-obtained individually tailored cue type and intercue distance, holographic visual cues were placed on a straight walk and a standardized FOG-evoking trail by the researcher.

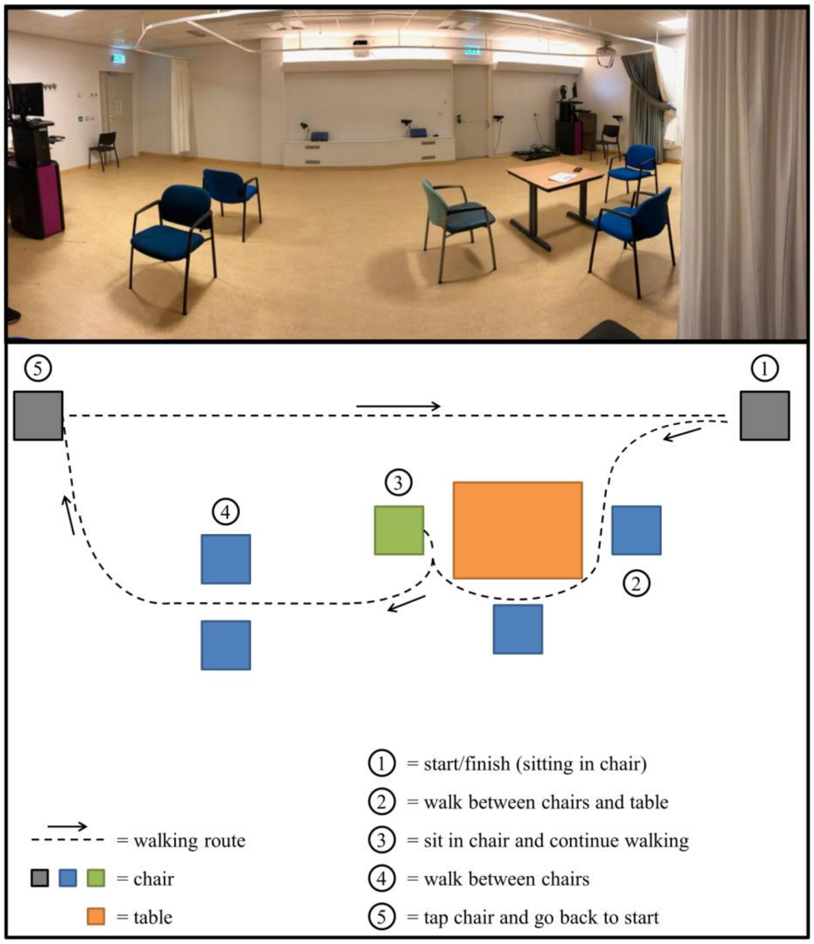

Figure S1. Standardized freezing-evoking trail in the ‘Technology in Motion’ lab.

Second, the participants were familiarized to walking with these holographic cues by letting them walk three times at a comfortable walking speed on a straight path with cues being continuously present. In addition, they were also familiarized to activating and deactivating the cues via specific voice commands. Participants then practiced this for approximately 5-10 minutes while walking a standardized FOG-evoking trail six times (Figure S1), including starting from standstill, turning, and walking through narrow apertures. This was performed twice at a comfortable walking speed, twice at a fast but safe walking speed, once at a comfortable walking speed while performing a cognitive dual task and once at a comfortable walking speed while holding a cup of water, leading to a total of six trials. Participants were instructed to 'turn the cues on and off when they wanted' without any specific instruction. The walking distance of the standardized FOG-evoking trail was about 20 m.

1.3 Session 3

Session 3 was again a home-based session to study habituation effects associated with wearing an unfamiliar device as well as the immediate effect of Holocue on FOG. Participants walked the same route in their house with the same number of trials (4-10) per condition and with the same instructions as in Session 1. They did so in two conditions: while wearing the HoloLens with and without the Holocue application for patient-tailored (according to Session 2) holographic cues (see Supplementary Video) with on-demand cue activation, in counter-balanced order. The instruction for both conditions was to 'walk the predetermined route at comfortable walking speed with/without cognitive dual task.'

Again, the researcher placed the holographic cues on the predetermined route using the intercue distance as determined in Session 2. Before starting with the Holocue condition, the participants had three practice trials with the cues being continuously present to experience where they might benefit from the cues. During the Holocue condition, participants were free to choose not to use the cues, use them continuously or activate the cues on demand upon freezing or preventively before they expected to freeze. The additional instruction was given to turn the cues on when they felt like they could help to reduce FOG and turn them off when they did not find them necessary. The researcher always walked closely behind the participant for safety reasons and walking trials were again filmed for offline annotation. The researcher also ensured similar walking routes, instructions and number of trials (including the number of trials with a cognitive dual task) per condition and over sessions. However, for five participants the number of trials did not agree between sessions due to physical condition limitations and trials of all conditions were therefore matched for fair between- and within-session comparisons of the number and duration of FOG episodes.

1.4 Structured semi-open standardized questionnaire

To evaluate acceptability of the Holocue application, a short questionnaire on usability (i.e., System Usability Scale; [11]) and a structured semi-open standardized questionnaire were filled out after completion of Session 3, in the absence of the researcher to avoid gratitude bias [12]. A total score on the System Usability Scale above 68 indicates that the usability of the system (i.e., HoloLens with Holocue application) was considered acceptable [11]. The structured questionnaire included elements of an existing questionnaire [13], evaluating user experience and acceptability with augmented-reality visual cues presented with smart glasses, and enquired about opportunities for improvement (see Supplementary Material 2 for the full questionnaire). Considering the known limitations of HoloLens 1 (rather uncomfortable bulky and heavy headset with a mixed-reality field of view limiting visibility of nearby holographic cues; [14]), questions also addressed acceptability of the

Holocue application with an improved headset in terms of comfort and field of view, as specified in Supplementary Material 2.

2 References

[1]. Goetz CG, Tilley BC, Shaftman SR, Stebbins GT, Fahn S, Martinez-Martin P, et al. Movement Disorder Society-sponsored revision of the Unified Parkinson's Disease Rating Scale (MDSUPDRS): scale presentation and clinimetric testing results. Mov Disord (2008) 23(15):2129-2170. doi: 10.1002/mds.22340

[2]. Hoehn MM, Yahr MD. Parkinsonism: onset, progression and mortality. Neurology (1967) 17(5):427-442. doi: 10.1212/wnl.17.5.427

[3]. Nasreddine ZS, Phillips NA, Bédirian V, Charbonneau S, Whitehead V, Collin I, et al. The Montreal Cognitive Assessment, MoCA: a brief screening tool for mild cognitive impairment. J Am Geriatr Soc (2005) 53(4):695-699. doi: 10.1111/j.1532-5415.2005.53221.x

[4]. Nieuwboer A, Rochester L, Herman T, Vandenberghe W, Emil GE, Thomaes T, et al. Reliability of the new freezing of gait questionnaire: agreement between patients with Parkinson's disease and their carers. Gait Posture (2009) 30(4):459-463. doi: 10.1016/j.gaitpost.2009.07.108

[5]. Ehgoetz Martens KA, Shine JM, Walton CC, Georgiades MJ, Gilat M, Hall JM, et al. Evidence for subtypes of freezing of gait in Parkinson's disease. Mov Disord (2018) 33(7):1174-1178. doi: 10.1002/mds.27417

[6]. Giladi N, Nieuwboer A. Understanding and treating freezing of gait in Parkinsonism. Proposed working definition and setting the stage. Mov Disord (2008) Supplement 23:423–425. doi: 10.1002/mds.21927

[7]. Lord SR, Bindels H, Ketheeswaran M, Brodie MA, Lawrence AD, Close JCT, et al. Freezing of gait in people with Parkinson's disease: nature, occurrence, and risk factors. J Parkinsons Dis (2020) 10(2):631-640. doi: 10.3233/JPD-191813

[8]. Spildooren J, Vercruysse S, Desloovere K, Vandenberghe W, Kerckhofs E, Nieuwboer A. Freezing of gait in Parkinson's disease: the impact of dual-tasking and turning. J Mov Disord (2010) 25(15):2563-2570. doi: 10.1002/mds.23327

[9]. Geerse DJ, Roerdink M, Marinus J, van Hilten JJ. Assessing walking adaptability in Parkinson's disease: "the Interactive Walkway". Front Neurol (2018) 9:1096. doi: 10.3389/fneur.2018.01096

[10]. Geerse DJ, Roerdink M, Marinus J, van Hilten JJ. Walking adaptability for targeted fall-risk assessments. Gait Posture (2019) 70:203-210. doi: 10.1016/j.gaitpost.2019.02.013

[11]. Fitzpatrick R, Hopkins A. Problems in the conceptual framework of patient satisfaction research: an empirical exploration. Sociol Health Illn (1983) 5:297-311. doi: 10.1111/1467-9566.ep10491836

[12]. Brooke, J. “SUS: a „quick and dirty“ usability scale”. In: Jordan PW, Thomas B, Weerdmeester BA, McClelland IL, editors. Usability Evaluation in Industry. London: Taylor and Francis (1996). p. 189-194.

[13]. Janssen S, Bolte B, Nonnekes J, Bittner M, Bloem BR, Heida T, et al. Usability of three-dimensional augmented visual cues delivered by smart glasses on (freezing of) gait in Parkinson’s disease. Front Neurol (2017) 8:279. doi: 10.3389/fneur.2017.00279

[14]. Janssen S, de Ruyter van Steveninck J, Salim HS, Bloem BR, Heida T, van Wezel RJA. The beneficial effects of conventional visual cues are retained when augmented reality glasses are worn. Parkinsons Dis (2020) 2020:4104712. doi: 10.1155/2020/4104712
