# Supplementary material for: Holocue: A Wearable Holographic Cueing Application for Alleviating Freezing of Gait in Parkinson's Disease
Source: Front Neurol. 2022 Jan 10;12:628388. doi: 10.3389/fneur.2021.628388 (PMC8784874; doi:10.3389/fneur.2021.628388)
Supplement: Supplementary Material 2 — The structured semi-open standardized questionnaire and individual scores on the closed-ended questions for the current (PART I) and future (PART II) Holocue application. [file Data_Sheet_2.PDF]

Supplementary Material 2

1 Individual scores on the closed-ended questions of the structured semi-open standardized questionnaire

Table S1. Individual scores on the closed-ended questions for the current Holocue application (1 = totally disagree, 5 = totally agree). Note that negatively phrased questions are transformed (i.e., all questions are framed positively) for easy interpretation. Questions corresponding to the column headers can be found in the questionnaire (section 2). Corresponding question numbers are presented before the header.

**Participant**	**System Usability Scale (1.1-1.10)**	***1.11* Recommend to friend**	***1.12* Take system home**	***1.13* Pleasant to use**	***1.14* Work as I want it to work**	***1.15* Satisfied**	***1.16* Comfortable**	***1.17* Cues clearly visible**	***1.18* Wear it on the street**	***1.19* Not more attention required**	***1.20* Could step over or on cues**	***1.21* Less freezing**	***1.22* Shorter episodes**	***1.23* Cues activated when I wanted**	***1.24* Walk better**
**1**	57.5	4	2	1	5	5	4	5	1	5	5	5	3	5	3
**2**	42.5	1	1	3	1	2	4	1	1	2	3	4	4	4	4
**3**	57.5	3	1	5	1	1	1	5	1	2	1	3	3	4	2
**4**	65.0	3	2	4	2	2	4	2	4	4	1	2	2	2	2
**5**	57.5	3	3	3	-	-	-	-	-	-	-	-	-	-	-
**6**	50.0	3	3	3	3	3	1	1	1	4	3	4	4	3	4
**7**	42.5	1	1	2	1	1	2	4	1	3	1	1	2	3	1
**8**	57.5	3	3	2	3	3	2	4	3	3	3	3	3	3	4
**9**	37.5	1	1	1	1	1	2	2	1	2	1	3	3	4	2
**10**	42.5	3	5	4	4	4	4	5	2	2	4	3	2	5	5
**11**	50.0	3	1	2	3	3	4	5	1	3	2	2	3	3	4
**12**	-	1	1	1	1	-	-	1	1	5	1	1	1	1	1
**13**	2.5	2	1	1	1	1	1	1	1	1	1	1	1	1	1
**14**	35.0	2	1	3	2	2	2	4	2	2	4	2	2	4	2
**15**	57.5	4	2	3	1	3	-	2	3	2	2	2	2	3	2
**16**	30.0	2	4	3	2	2	3	4	5	5	1	1	1	2	1
**17**	-	4	4	4	3	3	4	5	4	4	5	4	4	4	5
**18**	47.5	3	2	3	1	3	3	3	2	3	2	2	2	3	3
**19**	55.0	4	4	3	4	4	4	4	3	3	2	4	3	4	4
**20**	55.0	4	5	5	4	4	5	2	5	2	3	3	3	2	2
**21**	62.5	3	4	3	1	1	2	1	2	1	2	1	1	4	3
**22**	-	2	5	3	1	2	3	1	3	4	1	3	3	2	3
**23**	35.0	3	4	2	3	2	1	4	2	2	2	3	3	2	3
**24**	50.0	4	5	4	3	4	4	5	5	2	5	4	4	4	4
**avg.**	47.1	2.8	2.7	2.8	2.2	2.5	2.9	3.1	2.3	2.9	2.4	2.7	2.6	3.1	2.8

Table S2. Individual scores on the closed-ended questions for the future Holocue application (1 = totally disagree, 5 = totally agree). Negatively phrased questions are transformed for easy interpretation such that all questions are framed positively. Questions corresponding to the column headers can be found in the questionnaire (section 2). Corresponding question numbers are presented before the header.

**Participant**	***2.1* Recommend to friend**	***2.2* Open to new technologies**	***2.3* Holocue useful**	***2.4* I need Holocue**	***2.5* Want to use it home indoors**	***2.6* Want to use it home outdoors**	***2.7* Want to use it private outdoors**	***2.8* Want to use it in public**	***2.9* Expect less freezing**	***2.10* Expect shorter episodes**	***2.11* Important to choose type of cues**	***2.12* Important to step on cues**	***2.13* Important to step over cues**	***2.14* Important to choose color of cues**	***2.15* Important to choose size of cues**	***2.16* Important to choose distance between cues**	***2.17* Important to place cues on freezing-prone locations**	***2.18* More confident with Holocue**	***2.19* Walk better with cues**	***2.20* Auditory cues with visual cues**	***2.21* Auditory cues instead of visual cues**
**1**	5	5	5	2	4	4	4	4	5	5	5	5	1	3	5	5	5	4	5	3	5
**2**	3	4	4	3	3	3	3	3	4	4	2	4	3	4	4	5	5	4	4	2	3
**3**	2	4	4	1	1	1	1	1	1	1	5	2	4	3	4	4	4	2	2	5	1
**4**	4	3	4	2	1	3	2	3	3	3	5	5	5	3	4	4	5	4	3	3	3
**5**	4	4	4	2	2	4	2	3	3	3	2	2	2	2	2	2	2	2	2	4	2
**6**	5	5	5	4	4	4	5	5	5	5	3	5	5	5	5	5	5	5	5	5	5
**7**	2	5	3	3	2	2	2	1	1	1	3	3	3	3	3	3	3	3	1	4	3
**8**	4	4	4	3	3	4	4	4	4	4	4	4	3	3	3	4	4	4	4	4	5
**9**	4	4	4	2	4	4	4	2	4	2	4	4	4	4	4	4	5	2	4	4	4
**10**	4	4	4	3	4	4	2	2	4	4	4	4	3	3	3	3	3	4	4	1	1
**11**	4	3	3	3	4	4	3	3	5	5	5	5	5	5	5	5	5	5	5	3	3
**12**	1	1	1	1	1	1	1	1	1	1	1	3	1	1	1	1	1	1	1	3	3
**13**	1	5	1	1	1	1	1	1	5	5	5	5	5	5	5	5	5	5	2	3	4
**14**	2	4	2	2	2	2	2	2	3	2	4	4	2	3	4	4	4	2	2	2	2
**15**	4	4	3	2	3	3	3	3	2	2	3	3	3	3	3	3	3	3	3	3	3
**16**		5	4	1	2		2	1	1	1	5	5	5	5		1	1	4	2	3	5
**17**	4	5	5	4	4	4	4	3	5	5	5	3	3	3	4	5	4	4	4	3	3
**18**	3	4	4	3	4	4	4	4	4	4	4	4	4	4	4	4	4	4	4	2	2
**19**	4	4	4	3	2	4	4	4	4	4	4	3	3	4	4	4	4	4	4	3	4
**20**	2	3	2	2	2	2	2	2	2	2	4	4	3	4	4	4	4	2	3	4	2
**21**	4	5	4	2	2	3	3	3	4	4	4	4	3	4	3	4	4	4	3	4	4
**22**	4	5	3	3	5	4	5	3	4	4	5	5	4	3	3	5	4	5	4	2	2
**23**	3	4	4	3	4	4	4	4	3	3	5	4	4	3	4	4	4	3	3	3	4
**24**	4	5	5	3	3	5	5	5	5	5	3	3	3	3	5	5	4	4	5	3	3
**avg.**	3.3	4.1	3.6	2.4	2.8	3.2	3.0	2.8	3.4	3.3	3.9	3.9	3.4	3.5	3.7	3.9	3.8	3.5	3.3	3.2	3.2

2 Structured semi-open standardized questionnaire

PART I: USABILITY OF THE HOLOCUE APPLICATION

Instructions: *Below are a number of statements about the Holocue application. The statements relate to your experiences with using the headset with the Holocue application ("this system" in the questionnaire below) in its current form at home during the research. This information helps to identify of the strengths and possibilities for improvement of this system. For each question, please provide the answer that best fits your situation. If you are unsure how to answer a question, choose the option that most closely resembles your experience (one number per question). You can provide an explanation after question 24. The answers you provide will be shared anonymously with the researchers.*

		**Strongly disagree**	←	**Neutral**	→	**Strongly agree**
1.1	I think that I would like to use this system frequently	1	2	3	4	5
1.2	I found the system unnecessarily complex	1	2	3	4	5
1.3	I thought the system was easy to use	1	2	3	4	5
1.4	I think that I would need the support of a technical person to be able to use this system	1	2	3	4	5
1.5	I found the various functions in this system were well integrated	1	2	3	4	5
1.6	I thought there was too much inconsistency in this system	1	2	3	4	5
1.7	I would imagine that most people would learn to use this system very quickly	1	2	3	4	5

		Strongly disagree	←	Neutral	→	Strongly agree
1.8	I found the system very cumbersome to use	1	2	3	4	5
1.9	I felt very confident using the system	1	2	3	4	5
1.10	I needed to learn a lot of things before I could get going with this system	1	2	3	4	5
1.11	I would recommend this system to a friend	1	2	3	4	5
1.12	If I could take the system home I would	1	2	3	4	5
1.13	This system was pleasant to use	1	2	3	4	5
1.14	This system worked the way I would like it to work	1	2	3	4	5
1.15	I was satisfied with this system	1	2	3	4	5
1.16	This system was comfortable	1	2	3	4	5
1.17	The cues of this system were clearly visible	1	2	3	4	5
1.18	I would wear this system on the street	1	2	3	4	5
1.19	Walking with this system required more attention than walking without this system	1	2	3	4	5
1.20	I felt like I could step over or on the cues	1	2	3	4	5
1.21	I had fewer freezing episodes due to the cues of this system	1	2	3	4	5
1.22	I had shorter freezing episodes due to the cues of this system	1	2	3	4	5
1.23	This system activated the cues when I wanted	1	2	3	4	5
1.24	I walked better with this system	1	2	3	4	5

Optional explanation:

PART II: VALUE OF THE CONCEPT OF THE HOLOCUE APPLICATION AFTER TECHNICAL IMPROVEMENTS

This questionnaire will take approximately 15 minutes and is the last part of this study. The answers you provide are shared anonymously with the researchers.

Introduction: The development of the Holocue application is based on some assumptions about the value of the Holocue application for potential end users like you. It is important to test these assumptions with potential end users by means of statements about the Holocue application. Please answer as honestly as possible, whether it is positive or negative. It helps the researchers to better tailor the development of the Holocue application to the wishes and needs of potential end users.

You took part in one of the first studies with the Holocue application. It is known that the headset you have worn is not suitable for optimal use of the Holocue application. However, technology is developing fast. For example, the display in which the cues are presented will become larger, so that in the future the cues will be better visible at your feet. In addition, the headset will become lighter and smaller, which promotes future wearing comfort. The headset will also look more and more like glasses in the future. When answering the statements below, you are asked to take these future improvements in headset technology into account: greatly improved wearing comfort, larger field of view so that cues close to the feet are better visible, less obtrusive due to glasses-like appearance.

Propositions about the value of the Holocue application after technical improvements

With the Holocue application, visual cues can be activated preventively to prevent freezing. Visual cues can also be activated to alleviate a freezing episode. The cues are projected onto the walking surface via a headset and can be individually set in terms of color, size, distance and type. To what extent do you agree with the following statements, on a scale from 1 (strongly disagree) to 5 (strongly agree). You may comment on your answer if you wish.

		**Strongly disagree**	←	**Neutral**	→	**Strongly agree**
2.1	I would recommend the Holocue application to a friend	1	2	3	4	5

		Strongly disagree	←	Neutral	→	Strongly agree
2.2	I am open to new technologies such as the Holocue application	1	2	3	4	5
2.3	I find the Holocue application useful	1	2	3	4	5
2.4	I need the Holocue application	1	2	3	4	5
2.5	I want to use the Holocue application at home indoors	1	2	3	4	5
2.6	I want to use the Holocue application at home outdoors (for example in the garden)	1	2	3	4	5
2.7	I want to use the Holocue application in private situations elsewhere (for example with family)	1	2	3	4	5
2.8	I want to use the Holocue application in public situations (for example at the supermarket)	1	2	3	4	5
2.9	With the Holocue application I expect fewer freezing episodes	1	2	3	4	5
2.10	With the Holocue application I expect shorter freezing episodes	1	2	3	4	5
2.11	I think it is important that I can choose the type of cues myself	1	2	3	4	5
2.12	I think it is important that I can step on the cues	1	2	3	4	5
2.13	I think it is important that I can step over the cues	1	2	3	4	5
2.14	I think it is important that I can adjust the color of the cues myself	1	2	3	4	5

		Strongly disagree	⟵	Neutral	⟶	Strongly agree
2.15	I think it is important that I can adjust the size of the cues myself	1	2	3	4	5
2.16	I think it is important that I can set the distance between the cues myself	1	2	3	4	5
2.17	I think it is important that I can set the cues at locations where I often freeze	1	2	3	4	5
2.18	The Holocue application will give me more confidence in walking	1	2	3	4	5
2.19	The cues from the Holocue application make me walk better	1	2	3	4	5
2.20	I would like auditory cues <u>instead of</u> visual cues	1	2	3	4	5
2.21	I would like auditory cues <u>along with</u> visual cues	1	2	3	4	5

Optional explanation:

PART III: YOUR FEEDBACK

3.1 Do you think the researchers should continue or stop developing the Holocue application? Please explain your answer.

3.2 In case you advise the researchers to continue developing the Holocue application, what change(s) would you recommend?

3.3 Do you see any other possible uses of the headset or the Holocue application that you or others could benefit from? Please explain your answer.

3.4 This is the last question. Do you have any comments about the Holocue application or the research that you want to share with the researchers?

Thank you very much for participating in this study and for answering the questions. Your answers will be treated confidentially. This is the end of the questionnaire.
